# A Nomogram Based on Clinical and Ultrasound Characteristics to Predict Central Lymph Node Metastasis of Papillary Thyroid Carcinoma

**DOI:** 10.3389/fendo.2021.666315

**Published:** 2021-04-28

**Authors:** Jia-Wei Feng, Li-Zhao Hong, Fei Wang, Wan-Xiao Wu, Jun Hu, Sheng-Yong Liu, Yong Jiang, Jing Ye

**Affiliations:** Department of Thyroid Surgery, The Third Affiliated Hospital of Soochow University, Changzhou First People’s Hospital, Changzhou, China

**Keywords:** papillary thyroid carcinoma, central lymph node metastasis, central neck dissection, nomogram, surgery

## Abstract

**Background:**

The status of lymph nodes in the central compartment is crucial to determining the surgical strategies for papillary thyroid carcinoma (PTC). We aimed to develop a nomogram for predicting central lymph node metastasis (CLNM).

**Methods:**

A total of 886 PTC patients who underwent total thyroidectomy or lobectomy with central neck dissection (CND) from July 2019 to June 2020 were retrospectively retrieved. Clinical and ultrasound features were collected. Univariate and multivariate analysis were performed to determine risk factors of CLNM. A nomogram for predicting CLNM was developed, internal and external calibration was performed for the established model.

**Results:**

Variables (sex, chronic lymphocytic thyroiditis, tumor size, the number of foci, tumor location, margin) significantly associated with CLNM were included in the nomogram. The nomogram showed excellent calibration in the training group and validation group, with area under curves of 0.806 (95% CI, 0.771 to 0.825), and 0.799 (95% CI, 0.778–0.813) respectively.

**Conclusion:**

Through this accurate and easy-to-use nomogram, the possibility of CLNM can be objectively quantified preoperatively. Clinicians can use this nomogram to evaluate the status of lymph nodes in PTC patients and consider prophylactic CND for those with high scores.

## Introduction

The incidence of thyroid cancer is rising worldwide, and more than 90% of all thyroid cancers are differentiated thyroid cancer (DTC) (1). Papillary thyroid carcinoma (PTC) is the most common type of DTC, and tends to metastasize to cervical lymph nodes. Central compartment lymph nodes are the first to be involved in PTC. According to the American Thyroid Association Surgery Working Group, the central compartment refers to level VI. The VI region extends from the lower edge of the hyoid to the upper edge of the sternum, and the bilateral boundary is the bilateral common carotid artery. The central neck compartment is subdivided into four zones for the dissection: prelaryngeal (delphian), pretracheal, right and left paratracheal regions. As reported, the risk of lymph node metastasis (LNM) in the central neck compartment was the highest, ranging from 18% to 80% (2–4). Some studies reported that central lymph node metastasis (CLNM) was even associated with an increased risk of regional recurrence (5, 6).

Preoperative detection techniques such as high-resolution ultrasonography (US) and US-guided fine-needle aspiration (FNA) biopsy can greatly improve the diagnosis of PTC. However, due to the limitations of imaging technology, the detection rate of CLNM is relatively low before surgery. For example, the diagnostic sensitivity of US for CLNM is only 51% to 58.3%, and the false negative rate is as high as 44.6% (7, 8). Currently, there is no uniform standard to measure the advantages and disadvantages of routine central neck dissection (CND). Hence, there has been controversy about the role of routine CND. Under these circumstances, an appropriate and noninvasive tool that could quantify the risk of CLNM may be helpful for the optimal treatment of PTC patients.

Different from previous studies that only determine the risk factors of CLNM, we aimed not only to identify risk factors for predicting CLNM, but also to develop and validate the nomogram *via* clinical and US variables. Through this accurate and easy-to-use nomogram, which has excellent user-friendliness and convenience in formulating personalized treatments for patients, the possibility of CLNM can be objectively quantified preoperatively.

## Materials and Methods

### Patients

**Figure 1:**
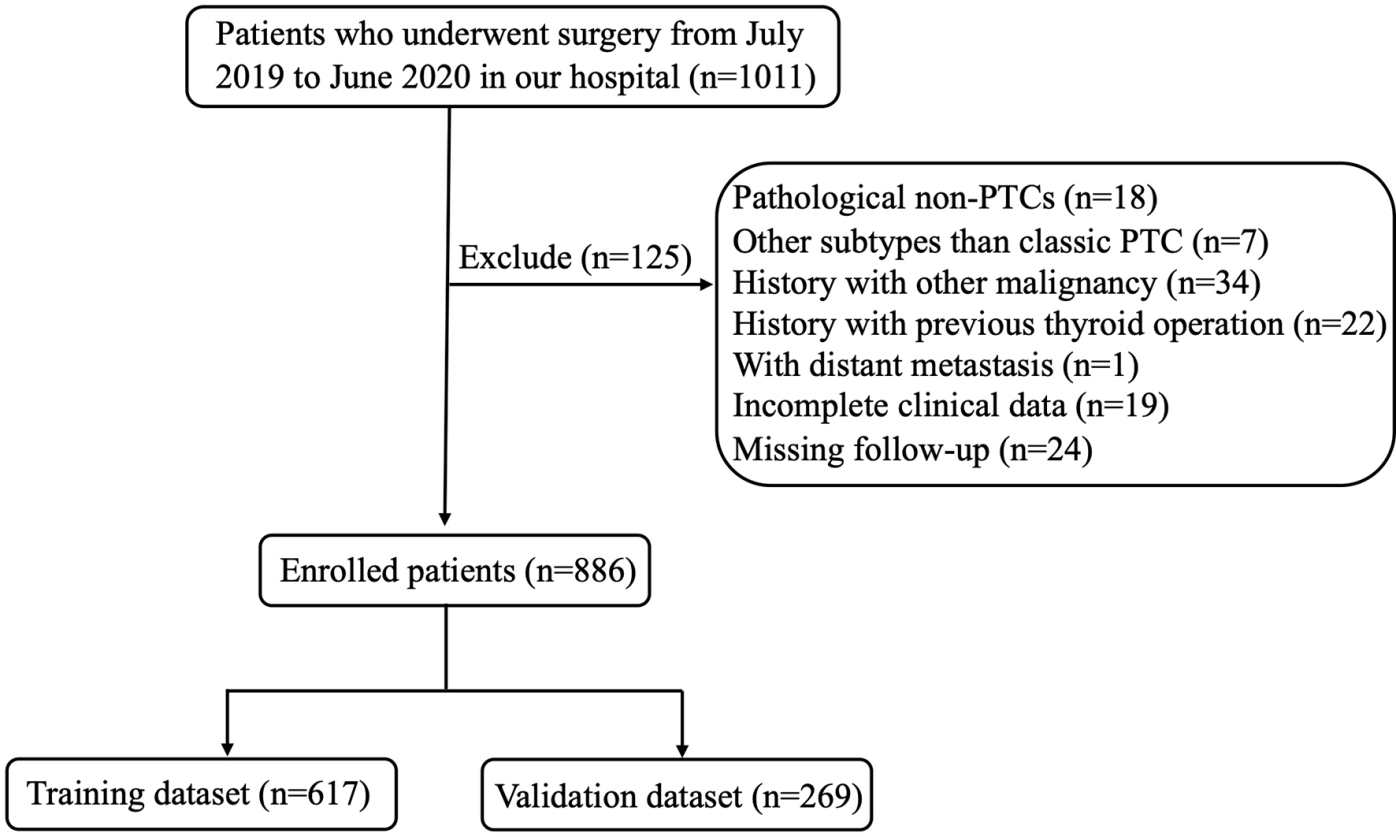
Flow chart of the patients enrolled in our study.

The study was approved by the Institutional Review Board of Changzhou First People’s Hospital. All participants gave written informed consent for their clinical records to be used in this study. The records of patients with PTC who underwent surgery from July 2019 to June 2020 at the Department of Thyroid Surgery of Changzhou First People’s Hospital were retrospectively reviewed. Patients were excluded from the study if they have any of the following factors: (1) non-PTCs (medullary/follicular/anaplastic) or other subtypes than classic PTC (such as mixed PTC and so on); (2) patients who underwent non-curable surgery or did not undergo CND; (3) patients with another malignancy before thyroidectomy; (4) patients with previous thyroid operation; (5) distant metastasis at diagnosis on pathological or clinical analysis; (6) history of neck radiation or familial cancer; (7) incomplete clinical data or missing follow-up. According to above criteria, 886 patients with PTC were enrolled in this study. **Figure 1** showed the flow chart of the patients enrolled in our study.

### Preoperative Examination and Surgical Procedures

With the discovery of thyroid nodule(s), a complete examination would be carried out. Apart from the routine measurement of thyroid hormone levels, US of the neck would be used to evaluate the primary lesions and cervical lymph nodes. We routinely conducted FNA to confirm the histopathologic diagnosis before surgery. The BRAF V600E mutation, which could help to diagnosis PTC, was also performed. Preoperative US characteristics of each nodule included the following features: aspect ratio (height divided by width on transverse views, A/T), tumor site (upper pole, upper part of the high plane of the isthmus; middle pole, parallel to the isthmus; and lower pole, lower part of the low plane of the isthmus), nodular composition (cystic or spongiform; mixed cystic and solid; solid), echogenicity (anechoic; hyperechoic or isoechoic; hypoechoic; very hypoechoic), margin (smooth; lobulated or irregular; extrathyroidal extension (ETE)), echogenic foci (none or large comet-tail artifacts; macrocalcifications; peripheral calcifications; punctate echogenic foci). Cervical lymph nodes were considered suspicious if they had one of the following characteristics: hyperechoic change, a round shape or necrosis, loss of the fatty hilum, microcalcifications.

Combined with FNA or imaging diagnosis, if patients have any of the following factors (tumor located in the thyroid isthmus, bilateral multifocality, tumor size >4.0 cm, or 1cm< tumor size ≤4.0 cm with risk factors of recurrence, presence of ETE), they would undergo the total thyroidectomy (TT). Otherwise, they would only undergo the lobectomy (9). CND was routinely performed in our institution. Bilateral CND was performed during TT, and ipsilateral CND was performed during lobectomy. TT was defined as the removal of two lobes, the isthmus, and the pyramidal lobe. Lobectomy was defined as the removal of the involved lobe, with the isthmus and the pyramidal lobe. The central compartment refers to level VI. Ipsilateral CND included the removal of prelaryngeal, pretracheal and ipsilateral paratracheal lymph nodes, whereas bilateral CND included the removal of prelaryngeal, pretracheal and bilateral paratracheal lymph nodes (10). All specimens were sent to the department of pathology for paraffin fixation and histological analysis.

### Pathological Examination

All pathology specimens were reviewed and cross-checked by two or more experienced pathologists microscopically. Two or more PTC foci within the thyroid was defined as multifocality. Two or more PTC foci in a single lobe were unilateral multifocality, while 2 or more PTC foci in both lobes or one lobe plus isthmus were bilateral multifocality. The diameter of the largest tumor focus was taken as the primary tumor size in multifocal tumors. Papillary thyroid microcarcinoma (PTMC) was defined as PTC ≤1.0 cm in its maximum diameter while macro-PTC was PTC >1.0 cm in its maximum diameter. The location of the tumor was determined by the largest dominant lesion when the patient had multifocal lesions. The location of the tumor was determined by the portion containing more than two-thirds of the tumor volume when the dominant lesion occupied 2 adjacent parts. We used a holistic definition of chronic lymphocytic thyroiditis (CLT) that included (i) elevated antibodies to thyroid peroxidase level, and/or (ii) findings of diffuse heterogeneity on US, and/or (iii) diffuse lymphocytic thyroiditis on histopathology to avoid selection bias (11).

### Statistical Analyses

All statistical analyses were performed using the SPSS v 25.0 software (Chicago, IL, USA), and R software version 3.5.3 (The R Foundation for Statistical Computing). Continuous variables were expressed as the means ± standard deviations (SD), categorical variables were reported as numbers and percentages. Patients were divided to a “training group” and “validation group” randomly. A t-test, Pearson’s chi-square test or Fisher’s exact test was used to compare the baseline characteristics of these two groups. Variables with a *P*<0.05 in the univariate analysis were included in the multivariate analysis, which were performed logistic regression analysis to assess risk factors for CLNM in PTC Patients. Variables with a *P*<0.05 in the multivariate analysis were then used to construct a risk prediction model – Nomogram, in R software. We used the receiver operating characteristic (ROC) curve to test the discriminative power and consensus of our established prediction model. The performance of the nomogram was further evaluated by the calibration chart, which plotted the predicted probability of the nomogram against the observed probability. According to our nomogram, the possibility of CLNM was quantified as a risk score, and each patient was divided into different subgroups through the calculated CLNM risk score. When there were total statistical differences between groups, Pearson’s chi-square test or Fisher’s exact test was used for pairwise comparison, and the *P* value of pairwise comparison was corrected by Bonferroni method.

## Results

### Baseline Clinical and US Characteristics of Patients With PTC

As summarized in **Table 1**, a total of 886 PTC patients including 205 males (23.1%) and 681 females (76.9%) underwent thyroidectomy plus CND in our institution. The average age at diagnosis was 43.4 ± 12.1 years (range from 23 to 77 years), the average BMI was 24.2 ± 4.57 kg/m^2^ (range from 11.13 to 38.67 kg/m^2^), and the average tumor size was 1.21 ± 0.92 cm (range from 0.11 to 8.53 cm). Diabetes was present in 59 patients (6.7%), and CLT was present in 274 patients (30.9%). A total of 714 patients (80.6%) were positive for BRAF V600E mutation, and 172 patients (19.4%) tested negative. Six hundred patients (67.7%) had solitary lesion, 184 patients (20.8%) had 2 foci, and 102 patients (11.5%) had 3 or more than 3 foci. Among 286 patients with multifocal lesions, 182 (20.5%) were confirmed to have bilateral multifocality, 104 (11.7%) were confirmed to have unilateral multifocality. Tumors located in the upper portion of the thyroid gland were detected in 296 (33.4%) patients, and tumors located in the middle/lower lobe of thyroid were detected in 590 (66.6%) patients. The detailed description of the tumor by US was shown in **Table 1**. There were 248 patients (28.0%) suspected of CLNM before surgery by US. And 437 (49.3%) were pathologically confirmed to have CLNM. The average number of removed lymph nodes in the central compartment was 7.8 ± 4.9 (range from 2 to 35); and the average number of metastatic lymph nodes was 2.6 ± 1.6 (range from 0 to 15). There were 737 patients (83.2%) with 6 or more lymph nodes removed during the operation and 149 patients (16.8%) with less than 6 lymph nodes removed during the operation.

**Table 1:** Baseline clinical and US imaging characteristics of patients with PTC.

Characteristics	Total	Training dataset	Validation dataset	*P* value
	n = 886	n = 617	n = 269	
Sex				
Male	205 (23.1%)	138 (22.4%)	67 (24.9%)	
Female	681 (76.9%)	479 (77.6%)	202 (75.1%)	0.410
Age (Y)				
Mean ± SD (range)	43.4 ± 12.1 (23–77)	43.5 ± 12.1 (25–77)	43.2 ± 11.9 (23–76)	0.994
≥55	151 (17.0%)	106 (17.2%)	45 (16.7%)	
<55	735 (83.0%)	511 (82.8%)	224 (83.3%)	0.870
BMI (kg/m^2^)				
Mean ± SD (range)	24.2 ± 4.57 (11.13–38.67)	24.10 ± 4.62 (13.13–37.27)	24.41 ± 4.47 (11.13–38.67)	0.930
Normal	481 (54.3%)	342 (55.4%)	139 (51.7%)	
Overweight	405 (45.7%)	275 (44.6%)	130 (48.3%)	0.302
Diabetes				
Absence	827 (93.3%)	575 (93.2%)	252 (93.7%)	
Presence	59 (6.7%)	42 (6.8%)	17 (6.3%)	0.789
BRAF V600E mutation				
Negative	172 (19.4%)	125 (20.3%)	47 (17.5%)	
Positive	714 (80.6%)	492 (79.7%)	222 (82.5%)	0.335
CLT				
Absence	612 (69.1%)	434 (70.3%)	178 (66.2%)	
Presence	274 (30.9%)	183 (29.7%)	91 (33.8%)	0.217
Maximum tumor size (cm)				
Mean ± SD (range)	1.21 ± 0.92 (0.11–8.53)	1.31 ± 1.10 (0.11–8.53)	1.23 ± 0.92 (0.12–7.90)	0.134
≤1	505 (57.0%)	348 (56.4%)	157 (58.4%)	
>1 to ≤2	255 (28.8%)	178 (28.8%)	77 (28.6%)	
>2 to ≤4	103 (11.6%)	74 (12.0%)	29 (10.8%)	
>4	23 (2.6%)	17 (2.8%)	6 (2.2%)	0.904
The number of foci				
1	600 (67.7%)	416 (67.4%)	184 (68.4%)	
2	184 (20.8%)	129 (20.9%)	55 (20.4%)	
3 or more	102 (11.5%)	72 (11.7%)	30 (11.2%)	0.956
Multifocality				
Solitary	600 (67.7%)	416 (67.4%)	184 (68.4%)	
Unilateral multifocality	104 (11.7%)	81 (13.1%)	23 (8.6%)	
Bilateral multifocality	182 (20.5%)	120 (19.4%)	62 (23.0%)	0.103
Location				
Upper	296 (33.4%)	203 (32.9%)	93 (34.6%)	
Middle/Lower	590 (66.6%)	414 (67.1%)	176 (65.4%)	0.628
Nodular composition				
Cystic or spongiform	2 (0.2%)	1 (0.2%)	1 (0.4%)	
Mixed cystic and solid	12 (1.4%)	9 (1.5%)	3 (1.1%)	
Solid	872 (98.4%)	607 (98.4%)	265 (98.5%)	0.777
Echogenicity				
Anechoic	3 (0.3%)	2 (0.3%)	1 (0.4%)	
Hyperechoic or isoechoic	45 (5.1%)	30 (4.9%)	15 (5.6%)	
Hypoechoic	816 (92.1%)	570 (92.4%)	246 (91.4%)	
Very hypoechoic	22 (2.5%)	15 (2.4%)	7 (2.6%)	0.971
A/T				
≤1	340 (38.4%)	237 (38.4%)	103 (38.3%)	
>1	546 (61.6%)	380 (61.6%)	166 (61.7%)	0.973
Margin				
Smooth	561 (63.3%)	400 (64.8%)	161 (59.9%)	
Lobulated or irregular	286 (32.3%)	193 (31.3%)	93 (34.6%)	
ETE	39 (4.4%)	24 (3.9%)	15 (5.6%)	0.276
Echogenic foci				
None or large comet-tail artifacts	320 (36.1%)	221 (35.8%)	99 (36.8%)	
Macrocalcifications	45 (5.1%)	33 (5.3%)	12 (4.5%)	
Peripheral calcifications	12 (1.4%)	9 (1.5%)	3 (1.1%)	
Punctate echogenic foci	509 (57.4%)	354 (57.4%)	155 (57.6%)	0.915
US-reported LN status				
Negative	638 (72.0%)	433 (70.2%)	205 (76.2%)	
Positive	248 (28.0%)	184 (29.8%)	64 (23.8%)	0.066
CLNM				
Negative	449 (50.7%)	311 (50.4%)	138 (51.3%)	
Positive	437 (49.3%)	306 (49.6%)	131 (48.7%)	0.806
No. of removed LNs in CC				
Mean ± SD (range)	7.8 ± 4.9 (2–35)	7.8 ± 4.9 (3–35)	7.7 ± 5.0 (2–32)	0.848
≥6	737 (83.2%)	516 (83.6%)	221 (82.2%)	
<6	149 (16.8%)	101 (16.4%)	48 (17.8%)	0.590
No. of metastatic LNs in CC, Mean ± SD (range)	2.6 ± 1.6 (0–15)	2.6 ± 1.7 (0–12)	2.5 ± 1.6 (0–15)	0.364

US, Ultrasound; PTC, papillary thyroid carcinoma; Y, year; SD, standard deviation; BMI, body mass index; CLT, chronic lymphocytic thyroiditis; ETE, extrathyroidal extension; LN, lymph node; CLNM, central lymph node metastasis; CC, central compartment.

There were 617 patients in the training dataset and 269 patients in the validation dataset. The training and validation groups had no significant differences in clinicopathological characteristics and US features of thyroid nodules (*P*>0.05 for all comparisons), which justified their use as training and validation cohorts.

### Clinical and US Factors Associated With CLNM in the Training Group

In the univariate analysis, CLNM presented the significant association with sex, CLT, tumor size, multifocality, the number of foci, tumor location, A/T, margin, echogenic foci (all *P*<0.05) (**Table 2**). We further conducted the multivariate logistic regression modeling to screen for significant variables associated with CLNM.

**Table 2:** Univariate analysis and multivariate analysis of factors associated with CLNM in the training dataset and score.

Characteristics	CLNM, No. (%)			Multivariate analysis		
	Presence (n = 306)	Absence(n = 311)	*P* value	Adjusted OR (95% CI)	*P* value	Score
Sex						
Female	212 (69.3%)	267 (85.9%)		1		0
Male	94 (30.7%)	44 (14.1%)	<0.001	2.753 (1.716–4.416)	<0.001	43
Age (Y)						
≥55	57 (18.6%)	49 (15.8%)				
<55	249 (81.4%)	262 (84.2%)	0.344			
BMI (kg/m^2^)						
Normal	165 (53.9%)	177 (56.9%)				
Overweight	141 (46.1%)	134 (43.1%)	0.455			
Diabetes						
Absence	288 (94.1%)	287 (92.3%)				
Presence	18 (5.9%)	24 (7.7%)	0.366			
BRAF V600E mutation						
Negative	66 (21.6%)	59 (19.0%)				
Positive	240 (78.4%)	252 (81.0%)	0.422			
CLT						
Presence	70 (22.9%)	113 (36.3%)		1		0
Absence	236 (77.1%)	198 (63.7%)	<0.001	1.877 (1.236–2.853)	0.003	24
Maximum tumor size (cm)						
≤1	124 (40.5%)	224 (72.0%)		1		0
>1 to ≤2	116 (37.9%)	62 (19.9%)		2.357 (1.866–4.403)	<0.001	33
>2 to ≤4	52 (17.0%)	22 (7.1%)		3.999 (2.735–5.847)	<0.001	67
>4	14 (4.6%)	3 (1.0%)	<0.001	5.342 (1.424–20.043)	0.013	100
The number of foci						
1	171 (55.9%)	245 (78.8%)		1		0
2	83 (27.1%)	46 (14.8%)		1.628 (1.196–3.106)	0.007	27
3 or more	52 (17.0%)	20 (6.4%)	<0.001	2.924 (1.564–5.468)	0.001	54
Multifocality						
Solitary	171 (55.9%)	245 (78.8%)		1		
Unilateral multifocality	58 (19.0%)	23 (7.4%)		1.341 (0.638–2.816)	0.439	
Bilateral multifocality	77 (25.2%)	43 (13.8%)	<0.001	4.610 (3.178–6.688)	0.344	
Location						
Upper	52 (17.0%)	151 (48.6%)		1		0
Middle/Lower	254 (83.0%)	160 (51.4%)	<0.001	3.604 (2.309–5.625)	<0.001	65
Nodular composition						
Cystic or spongiform	0 (0.0%)	1 (0.3%)				
Mixed cystic and solid	6 (2.0%)	3 (1.0%)				
Solid	300 (98.0%)	307 (98.7%)	0.294			
Echogenicity						
Anechoic	2 (0.7%)	0 (0.0%)				
Hyperechoic or isoechoic	19 (6.2%)	11 (3.5%)				
Hypoechoic	278 (90.8%)	292 (93.9%)				
Very hypoechoic	7 (2.3%)	8 (2.6%)	0.151			
A/T						
≤1	145 (47.4%)	92 (29.6%)		1		
>1	161 (52.6%)	219 (70.4%)	<0.001	1.343 (0.892–2.021)	0.158	
Margin						
Smooth	175 (57.2%)	225 (72.3%)		1		0
Lobulated or irregular	110 (35.9%)	83 (26.7%)		1.704 (1.205–2.410)	0.003	21
ETE	21 (6.9%)	2 (1.0%)	<0.001	2.330 (1.612–3.973)	<0.001	31
Echogenic foci						
None or large comet-tail artifacts	81 (26.5%)	140 (45.0%)		1		
Macrocalcifications	13 (4.2%)	20 (6.4%)		0.936 (0.378–2.315)	0.886	
Peripheral calcifications	6 (2.0%)	3 (1.0%)		0.938 (0.207–4.252)	0.934	
Punctate echogenic foci	206 (67.3%)	148 (47.6%)	<0.001	1.588 (1.051–2.400)	0.082	
No. of removed LNs in CC						
≥6	254 (83.0%)	262 (84.2%)				
<6	52 (17.0%)	49 (15.8%)	0.678			

Y, year; SD, standard deviation; BMI, body mass index; CLT, chronic lymphocytic thyroiditis; ETE, extrathyroidal extension; CLNM, central lymph node metastasis; LN, lymph node; CC, central compartment.

Multivariate analysis showed that male (OR: 2.735, 95% CI: 1.716–4.416, *P*<0.001), absence of CLT (OR: 1.877, 95% CI: 1.236–2.853, *P*=0.003), tumor size ranging between 1.0 and 2.0 cm (OR: 2.357, 95% CI: 1.866–4.403, *P*<0.001), tumor size ranging between 2.0 and 4.0 cm (OR: 3.999, 95% CI: 2.735–5.847, *P*<0.001), tumor size > 4.0 cm (OR: 5.342, 95% CI: 1.424–20.043, *P*=0.013), two tumor foci (OR: 1.628, 95% CI: 1.196–3.106, *P*=0.007), three or more tumor foci (OR: 2.924, 95% CI: 1.564–5.468, *P*=0.001), tumors located in the middle/lower pole (OR: 3.604, 95% CI: 2.309–5.625, *P*<0.001), lobulated or irregular margin (OR: 1.704, 95% CI: 1.205–2.410, *P*=0.003), and ETE (OR: 2.330, 95% CI: 1.612–3.973, *P*<0.001) remained independent predictive variables of CLNM, as shown in **Table 2**.

### Development of the Nomogram for Predicting CLNM in PTC Patients

**Figure 2:**
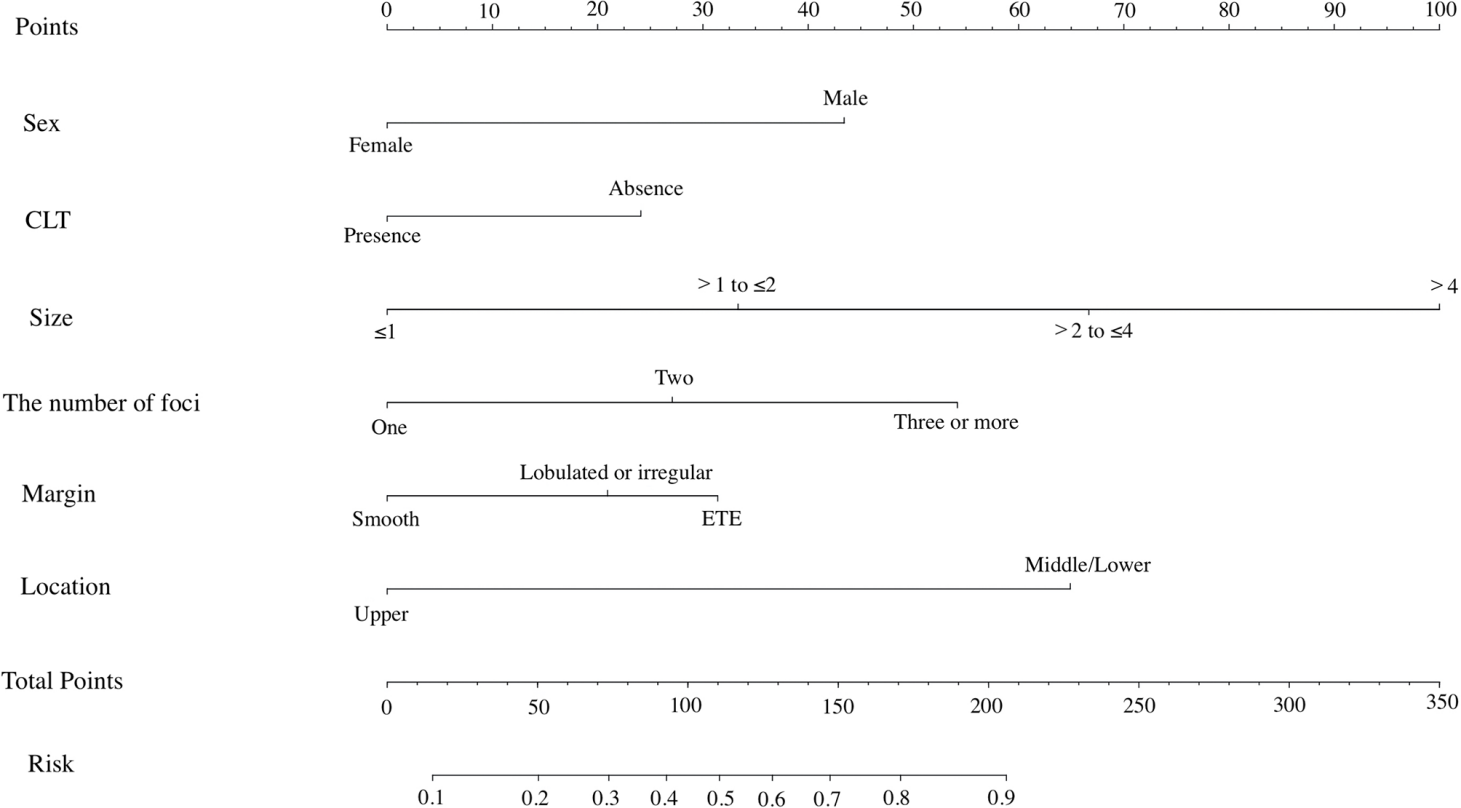
The nomogram for predicting CLNM in patients with PTC.

All risk factors that showed statistical significance in the logistic regression model were included in the nomogram, which could help estimate the metastasis risk of central compartment for individual patients with PTC (**Figure 2**). Each variable was proportionally assigned as the point on a scale from 0 to 100 in the nomogram based on the regression coefficient for CLNM. The nomogram confirmed tumor size as the largest contributor to scores. Detailed scores were listed in the **Table 2**. By adding the total score and positioning it on the scale of the total score, the corresponding probability of CLNM in each person can be determined.

### Validation of the Prediction Nomogram

**Figure 3:**
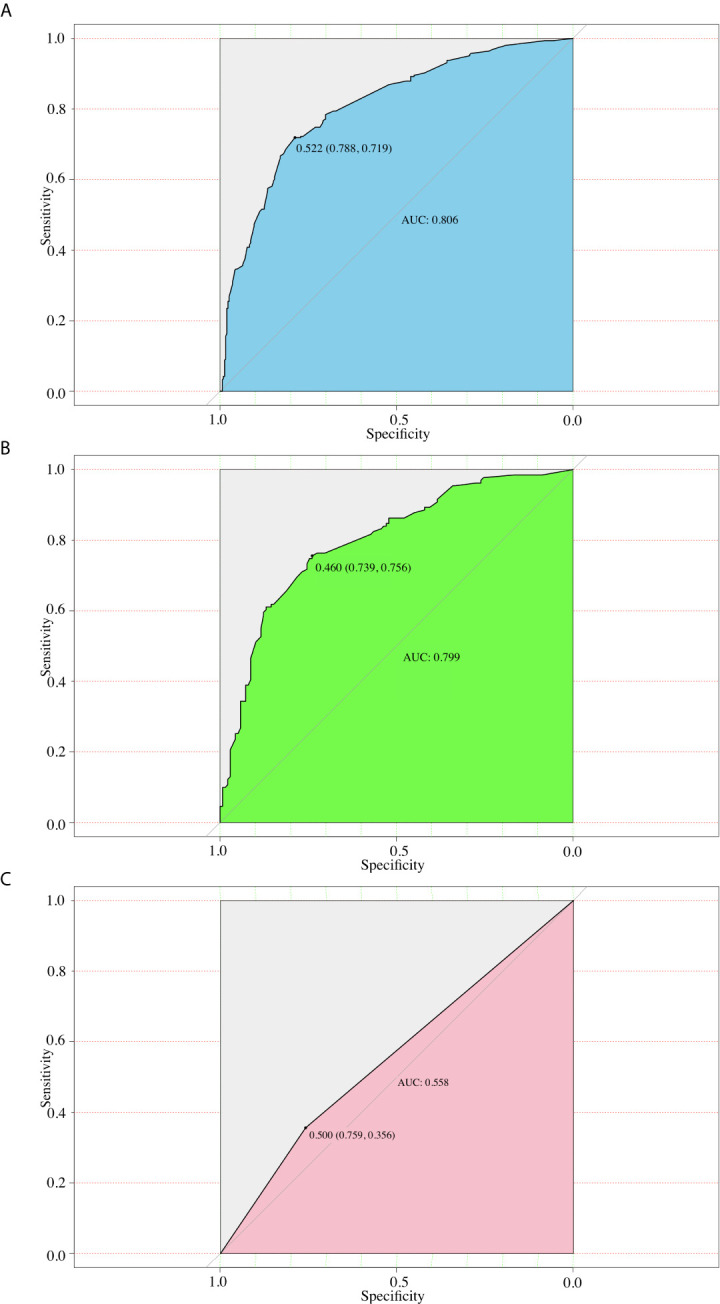
ROC curves for different models. **(A)** The ROC of the training group (AUC =0.806); **(B)** The ROC of the validation group (AUC =0.799); **(C)** The ROC of the preoperative US (AUC=0.558).

We then performed ROC analysis for the training and validation groups using this model (**Figures 3A, B**). The area under the curves (AUCs) in the training group and validation group were 0.806 (95% CI, 0.771 to 0.825), and 0.799 (95% CI, 0.778–0.813) respectively. Moreover, we calculated the AUC for preoperative US of predicting CLNM (**Figure 3C**). And the AUC was 0.558 (95% CI, 0.542–0.573) only, which was smaller than that of nomogram (*P*<0.001).

**Figure 4:**
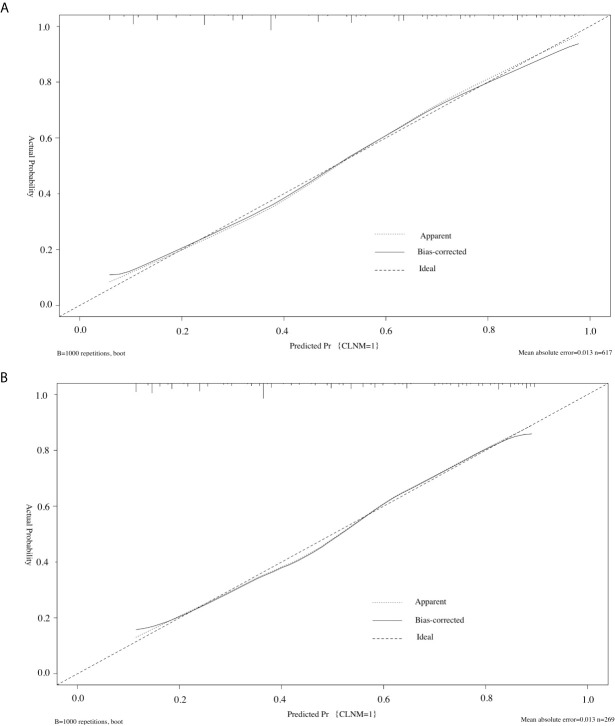
Calibration curve of the model in the training cohorts **(A)** and validation cohorts **(B)**. The diagonal dashed line represents the ideal prediction by the perfect nomogram; the solid line represents the calibration estimate from internally validated model; the dotted line indicates the apparent predictive accuracy. The closer the solid line is to the dotted line, the stronger the predictive ability of the model.

Furthermore, we used the similar bootstrap resampling procedure to conduct the internal and external calibration plot for the established model. Predicted and observed metastasis risks of CLNM were in good agreement. Moreover, the corrected risks also showed excellent agreement with observed metastasis risk after the adjustment for optimism, and only minor discrepancies were observed (**Figures 4A, B**).

### Novel Risk Stratification Based on the Predictive Nomogram

Considering that each variable contained in the nomogram has its corresponding risk point, and the total risk score calculated for all patients can quantitatively predict their respective CNM risk, we thereby determined three cut-off values (50, 100, 150) by using recursive partition analysis. As shown in **Table 3**, we established four subgroups as follows: (1) extreme low-risk group (patients with the nomogram score of ≤ 50), (2) low-risk group (50 < risk score ≤ 100), (3) moderate-risk group (100 < risk score ≤ 150), and (4) high-risk group (patients with the score of >150). In the training group, the rates of CLNM for extreme low, low, moderate, and high-risk groups were 12.6%, 29.7%, 62.1%, and 82.9%, respectively (*P*<0.001). Similarly, in the validation group, the rates of CLNM for extreme low, low, moderate, and high-risk groups were 12.0%, 31.6%, 60.3%, and 83.6%, respectively (*P*<0.001). We further studied whether the relative risk for CLNM in each risk category identified by the nomogram were significantly different from each other. After paired comparison, we found there were significant differences between all groups.

**Table 3:** Metastasis risk stratification of patients with PTC based on risk scores of nomogram model.

Nomogram	ELR	LR	MR	HR	Total	Total *P* value	ELR-LR	ELR-MR	ELR-HR	LR-MR	LR-HR	MR-HR
	0-50	51-100	101-150	>150			*P* value	*P* value	*P* value	*P* value	*P* value	*P* value
Training dataset												
Without CLNM	90 (87.4%)	128 (70.3%)	66 (37.9%)	27 (17.1%)	311							
With CLNM	13 (12.6%)	54 (29.7%)	108 (62.1%)	131 (82.9%)	306	<0.001	0.001	<0.001	<0.001	<0.001	<0.001	<0.001
Total	103	182	174	158	617							
Validation dataset												
Without CLNM	44 (88.0%)	54 (68.4%)	29 (39.7%)	11 (16.4%)	138							
With CLNM	6 (12.0%)	25 (31.6%)	44 (60.3%)	56 (83.6%)	131	<0.001	0.011	<0.001	<0.001	<0.001	<0.001	0.002
Total	50	79	73	67	269							

PTC, papillary thyroid carcinoma; CLNM, central lymph node metastasis; ELR, extreme low risk; LR, low risk; MR, moderated risk; HR, high risk.

## Discussion

With the increasing incidence of thyroid cancer, surgical resection is generally considered to be the most effective treatment for PTC. Decisions regarding the extent of surgery for the patient with PTC are mainly based on the preoperative assessment of lymph node status. But the role of prophylactic CND for clinically lymph node-negative (cN0) patients with PTC is still under debate. Supporters pointed that prophylactic CND not only eliminated potential recurrent sources, thereby reducing the risk of reoperation, but also improved the accuracy of staging (12, 13). Considering the potential complications of prophylactic CND, such as permanent hypoparathyroidism, recurrent laryngeal nerve injury and so on, opponents hold the view that prophylactic CND had the low prognostic benefits and many surgeons worldwide still preferred therapeutic CND only (14, 15). For cN0 PTC patients, the incidence of CLNM detected by histopathological examination ranged from 31% to 60.9% according to previous reports (16, 17). Therefore, routine CND is preferred for patients with PTC in our country due to the high risk of CLNM and unreliability of preoperative examinations in detecting CLNM.

The incidence of CLNM in our study was 49.3%, which was in accordance with the data of 24% to 58% reported in other studies (18, 19). We aimed to develop a nomogram, which could behave as a novel strategy to personalize and quantify the probability of CLNM in patients with PTC. Although some previous studies have also attempted to develop nomograms to predict CLNM for PTC patients, there were some limitations. For example, despite a nomogram with good discrimination (AUC=0.764) was built by Thompson et al. (13), only four variables were considered in this nomogram, which limited the clinical guidance. Moreover, these results were not reproducible in the external validation (AUC=0.615). Based on the 845 cN0 PTC patients with tumor size larger than 2 cm, Lang et al. (20) developed a nomogram, which showed a low discrimination (AUC=0.69) and was not validated in this study. Although enrolled larger patient cohorts, the AUC of 0.711 was not high for the nomogram established by Wang et al (21). In our study, we not only evaluated a large number of PTC patients, but also conducted both internal and external verification.

According to our findings, sex, CLT, tumor size, the number of foci, tumor location, margin were independent risk factors of CLNM among PTC patients by both univariate and multivariate analysis. Many clinicopathological factors related to CLNM have been reported previously, including sex (22–24), tumor size (13, 25), location (26), ETE (27), and the number of foci (28, 29). The incidence of PTC in women was significantly higher than that in men, and the ratio of women to men was approximately 3.7:1. However, the rate of CLNM in men was significantly higher than that in women (22–24). The relationship between multifocality and CLNM remains controversial (30). We divided the multifocality into unilateral multifocality and bilateral multifocality according to the location of tumors, and we found multifocality was not the independent risk factor of CLNM by multivariate logistic regression analysis. Instead of limited to investigating the difference between solitary and multifocal tumors, we further investigated the significance of the number of tumor foci on the incidence of CLNM. We found the proportion of CLNM increased with the number of foci, which was consistent with the study of Afif et al. (28) and Qu et al. (29). PTC cells from the upper region are more likely to be transported to the lateral lymph nodes through the lymph flow along the superior thyroid artery (31). Hence, tumor located in the upper pole of the thyroid lobe conferred a lower risk of CLNM and a higher risk of lateral cervical metastasis. It was known that larger tumor size was associated with more aggressive features in PTC. Interestingly, tumor size was the largest contributor to scores in our nomogram. Considering the guiding role of the nomogram before surgery, we took ETE diagnosed by preoperative US as variable instead of pathological ETE in this study. As reported, US showed high sensitivity (80%) for predicting minimal ETE of PTC (32). In addition, when US and magnetic resonance imaging (MRI) are combined, the diagnostic value of preoperative prediction of ETE would be greatly improved. Since our study is a retrospective study, MRI examinations were not performed for all patients. But we found lobulated or irregular margin and ETE detected by US were independent risk factors of CLNM. The pathogenetic mechanisms linking CLT and PTC are still poorly understood since1955 when the association between CLT and PTC was first proposed. Some mechanisms, such as elevated thyroid-stimulating hormone, RET/PTC rearrangement, and promoting tumor inflammation, have been proposed to explain the association between CLT and PTC (33). Our results showed that CLT was a protective factor against CLNM in PTC patients, which was in agreement with the meta-analysis of Lee et al. (34), that the lymphocytic infiltration counteracted tumor progression. Because the punctate echogenic foci were the strongest predictor of PTC in the US characteristics, the potential impact of microcalcification on CLNM should be discussed. In our study, echogenic foci were associated with the CLNM in the univariate analysis. But echogenic foci, especially punctate echogenic foci, were not the independent risk factors of CLNM by multivariate analysis. This may be due to other pathological structures, such as focal fibrosis of nodular goitres, which look similar to microcalcifications on US.

We incorporated the US characteristics and clinical risk factors into this easy-to-use nomogram, which may help individualized prediction of CLNM before surgery. The usage of nomogram is as follows: locate the patient’s sex on the sex axis. Draw a line straight upward to the point axis to establish how many points toward the probability of CLNM the patient may get. Repeat the process for each of the other variables. Calculate total points for each of the predictors. Pinpoint the final score on the total point axis. Draw a line straight down to determine the patient’s predicted probability of CLNM. For example, nomogram predicted a PTC male (43 points) patient with only one tumor (0 point) located in the middle portion (65 points), without CLT (24 points). According to US, the tumor had irregular margin (21 points), the size of tumor was 1.5cm (33 points). The total point was 186 for this patient. This patient had more than 80.0% chance of CLNM. By comparison with preoperative US, this nomogram showed a significant advantage over preoperative US (**Figure 3**). Apart from identifying the existence of CLNM, nomogram could also be used to guide surgeons to stratify patients so as to avoid unnecessary surgery. Based on the predictive nomogram, we proposed a risk stratification scheme and divided PTC patients into four quantified risk stratification (**Table 3**). For patients with different ratings, we can offer different treatment options. For example, for patients with extreme low risk or low risk of CLNM, prophylactic CND should be avoided to reduce surgical complications and damage; for patients with moderated risk of CLNM, prophylactic CND can be considered; for patients with high risk of CLNM, prophylactic CND is highly recommended to reduce the incidence of recurrence. In addition, for PTC patients who have not undergone CND, our nomogram may be helpful in detecting residual CLNM.

Despite some encouraging results were achieved, this study still had some limitations, which we would address in future studies. First, our study is a retrospective study. Compared with prospective studies, retrospective studies tend to have more errors and biases. For example, the criteria used to evaluate the US signature were subjective. Sonographers with insufficient experience may cause errors in a small sample. Nevertheless, the consensus of each feature among the sonographers in our study was consistent. The data we provided were extracted from the document and were not captured in the actual conversation. This model could also be improved by adding more useful technological parameters such as elastography and computer-aided diagnosis system. Second, the validation of the nomogram might be biased by institutional diagnostic patterns. Hence, strict external verification is required in prospective multi-center institutional trials to obtain more objective conclusions. Moreover, different surgeons were involved in performing thyroidectomy and lymph node dissection. Postoperative results, such as the number of metastasized lymph nodes may be affected by surgeon-specific factors.

In conclusion, our study found that CLNM was independently associated with sex, CLT, tumor size, the number of foci, tumor location, and margin. By using above variables, we constructed a nomogram that stratifies PTC patients into four groups that possess different CLNM risk levels. Clinicians can use these nomograms to evaluate the status of lymph nodes in PTC patients and consider prophylactic CND and meticulous postoperative evaluation for those with high scores.

## Data Availability Statement

The raw data supporting the conclusions of this article will be made available by the authors, without undue reservation.

## Ethics Statement 

This study has been approved by the Institutional Review Board of Changzhou First People’s Hospital ethics committee, and has been performed according to the ethical standards laid down in the 1964 Declaration of Helsinki. Written informed consent was obtained from all individual participants included in the study.

## Author Contributions

J-WF and L-ZH: writing - original draft, software, and data curation. S-YL: validation, formal analysis, and data curation. W-XW: conceptualization. FW and JH: validation and investigation. JY and YJ: writing - review & editing, visualization, and supervision. All authors contributed to the article and approved the submitted version.

## Funding

This research did not receive any specific grant from funding agencies in the public, commercial, or not-for-profit sectors.

## Conflict of Interest

The authors declare that the research was conducted in the absence of any commercial or financial relationships that could be construed as a potential conflict of interest.
